# A longitudinal census of the bacterial community in raw milk correlated with *Staphylococcus aureus* clinical mastitis infections in dairy cattle

**DOI:** 10.1186/s42523-022-00211-x

**Published:** 2022-11-24

**Authors:** Soyoun Park, Dongyun Jung, Ianina Altshuler, Daryna Kurban, Simon Dufour, Jennifer Ronholm

**Affiliations:** 1 Faculty of Agricultural and Environmental Sciences, Macdonald Campus, McGill University, Montreal, QC Canada; 2 Faculté de Médecine Vétérinaire, Université de Montréal, Saint-Hyacinthe, QC Canada; 3 Mastitis Network, Saint-Hyacinthe, QC Canada; 4 Regroupement FRQNT Op+Lait, Saint-Hyacinthe, QC Canada

**Keywords:** *Staphylococcus aureus*, Bovine mastitis, Microbiome, Bacterial interactions

## Abstract

**Background:**

*Staphylococcus aureus* is a common cause of clinical mastitis (CM) in dairy cattle. Optimizing the bovine mammary gland microbiota to resist *S. aureus* colonization is a growing area of research. However, the details of the interbacterial interactions between *S. aureus* and commensal bacteria, which would be required to manipulate the microbiome to resist infection, are still unknown. This study aims to characterize changes in the bovine milk bacterial community before, during, and after *S. aureus* CM and to compare bacterial communities present in milk between infected and healthy quarters.

**Methods:**

We collected quarter-level milk samples from 698 Holstein dairy cows over an entire lactation. A total of 11 quarters from 10 cows were affected by *S. aureus* CM and milk samples from these 10 cows (n = 583) regardless of health status were analyzed by performing 16S rRNA gene amplicon sequencing.

**Results:**

The milk microbiota of healthy quarters was distinguishable from that of *S. aureus* CM quarters two weeks before CM diagnosis via visual inspection. Microbial network analysis showed that 11 OTUs had negative associations with OTU0001 (*Staphylococcus*). A low diversity or dysbiotic milk microbiome did not necessarily correlate with increased inflammation. Specifically, *Staphylococcus xylosus*, *Staphylococcus epidermidis*, and *Aerococcus urinaeequi* were each abundant in milk from the quarters with low levels of inflammation.

**Conclusion:**

Our results show that the udder microbiome is highly dynamic, yet a change in the abundance in certain bacteria can be a potential indicator of future *S. aureus* CM. This study has identified potential prophylactic bacterial species that could act as a barrier against *S. aureus* colonization and prevent future instances of *S. aureus* CM.

**Supplementary Information:**

The online version contains supplementary material available at 10.1186/s42523-022-00211-x.

## Introduction

Bovine mastitis, a mammary gland inflammation mainly caused by bacterial infection, is one of the most prevalent and costly diseases in dairy cattle and has a significant impact on the profitability of the dairy industry, animal welfare, antimicrobial use, and public health. Bovine mastitis costs the dairy industry approximately $2 billion in the USA, about CAD$794-million in Canada, and £168 million in the UK annually [1–3]. Bacteria, the most common cause of bovine mastitis, include a broad range of environmental and contagious pathogens, encompassing more than 137 species [4]. Furthermore, the absent of the mucosal layer in the mammary gland directly exposes the epithelium to all resident bacteria [5]. *Staphylococcus aureus* is a common etiological agent of contagious bovine mastitis that can be responsible for either subclinical mastitis (SCM) or clinical mastitis (CM); although, knowledge gaps persist and influence diagnosis, treatment, and prevention [6–8]. Bovine specific pathoadaptive clonal lineages of *S. aureus* have emerged and spread alongside the use of antimicrobials in the dairy industry – increasing the prevalence of antimicrobial resistance in these lineages [9]. A recent study on the resistome of bovine clinical mastitis microbiome has revealed that *S. aureus* isolates had the highest resistance to several antibiotics such as doxycycline, ampicillin, tetracycline, and erythromycin [10]. More recently, antibiotics, especially high priority category I and II antibiotics, have been banned or highly regulated in agriculture to reduce the dissemination of antibiotics resistant genes into human pathogens [9, 11, 12]. The withdrawal of antimicrobials raises other concerns such as farm productivity and the prevalence of infectious diseases in livestock. Thus, alternatives to antimicrobials are required to support sustainable agriculture [13].

The microbiome plays a fundamental role in maintaining host health by metabolizing indigestible nutrients, biosynthesizing vitamins, educating the immune system, and providing microbial defences to the outgrowth of pathogens [14, 15]. It is now well accepted that commensal and symbiotic bacteria inhabiting the host have a potential role in resilience to exogenous perturbances. Thus, targeting and modulating the microbiome have been suggested as a promising alternative for mastitis prevention and treatment [16, 17]. Several studies on the oral administration of probiotics in breastfeeding women have shown the efficacy of probiotics for human mastitis prevention and treatment while its effectiveness on bovine mastitis is still unclear [18–20]. Intramammary probiotics or their infusion to dairy cows also remains questionable due to pro-inflammatory effects [21–24]. Despite the absence of evidence supporting the effectiveness of probiotics to prevent or treat bovine mastitis, the use of probiotics and their active biomolecules remains an area of interest for the development of alternative prophylactics and therapeutics [17, 25].

Studies examining the microbiota of the bovine udder and raw milk have shown the presence of a diverse and dynamic microbial community [15, 26, 27]. Ganda et al. (2016) showed reduced species diversity in raw milk collected from quarters with *Escherichia coli* CM infection compared to those from healthy quarters in 40 cows [28]. Another study showed that infected quarters (n = 28) were frequently dominated by a single operational taxonomic unit (OTU) [27]. There have been inter-study differences in the microbial changes in post-mastitic milk. Falentin et al. observed long-lasting microbiome perturbations in quarters with a history of clinical mastitis in the previous lactations while Ganda et al. reported the restoration of the microbiota 14 days after diagnosis of mastitis [29, 30]. However, it is unknown if the disruption in the microbial diversity occurs because of a CM infection, or if the microbial changes can be detected prior to the infection and play a role in susceptibility to CM.

In this study, we hypothesize that the composition and level of diversity seen in the bacterial community of raw milk is predictive of which quarters will develop *S. aureus* CM, and that the presence of certain bacteria may be negatively correlated with the presence of *S. aureus*. We aimed to understand the longitudinal changes in the bacterial community composition in both healthy and sick quarters before, during, and after *S. aureus* CM and to identify specific bacterial taxa that are negatively correlated with colonization by *S. aureus* and may, therefore, have an antagonistic relationship with this important pathogen.

## Materials and methods

### Milk sample collection

A total of 698 Holstein dairy cows from Canadian dairy herds located in the province of Quebec, in proximity to the Faculty of Veterinary Medicine of Université de Montréal (Saint-Hyacinthe) were enrolled in the project. Quarter level milk samples were collected every other week from the recruited cows before dry-off and following parturition as well as during lactation, between December 2018 and February 2020, from five different dairy herds. All milk samples were collected aseptically according to the recommended instruction by the Mastitis Network (http://www.reseaumammite.org/tactic/fr/echantillonnage/). More than 27,000 individual milk samples were collected by the research staff during this study period and kept between − 10 °C and − 20 °C due to the limited cold storage space; although, rapid freezing at − 80 °C would have been ideal [31]. Producers (daily) and research staff (during every other week sampling visit) identified CM via visual inspection of the milk and udder. Somatic cell count (SCC) was measured on most non-clinical milk samples. Microbiological culture of all milk samples was conducted by spreading 10 µL of raw milk on 5% sheep blood agar [32]. After a 24 to 48-h incubation period at 35 °C, the number of different bacterial phenotypes observed on the agar were enumerated. Milk samples harboring three or more dissimilar colony types on blood agar were considered contaminated according to national mastitis council recommendation [33]. On non-contaminated samples, colonies were enumerated, and a colony representative of each phenotype (1 or 2 phenotypes) was analyzed using matrix-assisted laser desorption/ionization time-of-flight (MALDI-TOF) mass spectrometry to identify the etiological agents [34]. Mammary quarters with CM and from which *S. aureus* was isolated in pure or mixed culture, including samples that produced > 3 types of colony morphology on blood agar, were infected by *S. aureus*. Among the 166 quarters from 135 cows diagnosed with CM during our study period, 11 quarters from 10 cows were diagnosed with *S. aureus* CM. From those 10 cows, a total of 599 milk samples were collected from all 40 quarters (infected and not infected) every two weeks preceding and following *S. aureus* CM as well as on the CM diagnosis day (Additional file 1: Fig. S1). The naming convention used, for example H1C120, is indicative of heard number (H) and cow number (C). The naming conventions for each milk sample included: collection date (YYMMDD), the assigned cow number (C) and a quarter (Q).

### DNA extraction

Milk samples were thawed on ice and mixed thoroughly by inverting the tubes. A 1.0 mL aliquot of milk was used for DNA extraction. Each milk sample was centrifuged at 16,000×*g* for 10 min and then the supernatant was discarded. For 16S rRNA gene amplicon sequencing, bacterial DNA was extracted from the remaining pallet via bead beating using DNeasy® PowerFood® Microbial Kit (QIAGEN, Germany) in combination with the QIACube instrument (QIAGEN, Germany) following the manufacturer’s instructions. Bacterial DNA from milk samples with Good’s coverage < 99.0% after sequencing were re-extracted and re-sequenced. An independent negative extraction control, which included extracting DNA from DNA/RNA free water using each of the reagents present in the extraction kit, was performed for each kit used in this study. A positive extraction control, which included total DNA extracted from a generous donor (GD) bovine rumen sample, was also performed using each DNA extraction kit used in this study, and results from each kit were compared to verify consistency in the study. For shotgun metagenomic sequencing, bacterial DNA was extracted using the same kit with 1.0 to 6.0 mL of milk, and the extracted DNA was then cleaned up using DNeasy® PowerClean® Pro Cleanup Kit (QIAGEN, Germany). The concentration and purity of DNA were evaluated using Invitrogen™ Quant-iT™ dsDNA Assay Kit (Thermo Fisher Scientific, USA) and a Nanodrop 2000 (Thermo Scientific, USA).

### PCR amplification, library preparation, and high throughput 16S rRNA gene amplicon sequencing

Milk samples (n = 593) were analyzed by 16S rRNA gene amplicon sequencing (Additional file 2: Table S1). Illumina MiSeq paired end (2 × 250 bp) sequencing was used to determine the bacterial community of each milk sample. The V4 hypervariable region of the bacterial 16S rRNA gene was amplified using the F548 and R806 primer pair [35]. The PCR was performed with denaturation at 95 °C from 5 min, 35 cycles of amplification (95 °C for 30 s, 50 °C for 30 s, and 72 °C for 1 min), and one final extension cycle at 72 °C for 10 min using HotStartTaq® *Plus* Master Mix Kit (QIAGEN, Germany). An independent negative PCR control, which consisted of an attempt to amplify DNA/RNA free water, was included for each 96-well PCR reaction performed as part of this study and subjected to sequencing. The amplicons were purified using Agencourt AMPure® XP (Beckman Coulter, Brea, CA, USA) and quantified with Invitrogen™ Quant-iT™ dsDNA Assay Kit. The DNA was pooled at equimolar concentration prior to the sequencing and then the pooled library was sequenced using the MiSeq and the MiSeq reagent kit V2 (Illumina Inc., USA) for 500 cycles (251 × 2).

### Shotgun metagenomic library preparation and high-throughput sequencing

Milk samples (n = 3) were identified as being of interest for additional analysis, based on the results of 16S rRNA gene amplicon sequencing, because of a microbiota highly dominated by a single taxonomic group with the low SCC (< 200,000 cells/mL). These samples were further analyzed via shotgun metagenomics. Sequencing libraries were prepared with Nextera XT DNA Flex Library Preparation Kit (Illumina Inc., USA) and Nextera XT Index Kit (Illumina Inc., USA) according to the manufacturer’s instructions. Paired-end sequencing (2 × 150 bp) was performed on a NovaSeq 6000 machine (Illumina Inc., USA) at Genome Quebec (Montreal, Canada).

### 16S rRNA gene amplicon sequencing data analysis

The FASTQ files obtained from the MiSeq sequencer were analyzed using Mothur (v. 1.42.3) [36]. OTU picking was performed using the SILVA v138.1 database [37]. Good’s coverage was calculated and performed using MicrobiomeAnalyst [38]. Sequences were rarefied (vegan::rarefy.perm) repeatedly 1000 times to minimum number of sequences (n = 3068) to obtain the average rarefied OTU table with vegan R package (v. 2.6–2) [39–41], which was then used for further analysis. Alpha-diversity (Chao1, Shannon, and Simpson) was calculated and Mann–Whitney test (no paired) was performed with vegan R package. Beta-diversity (Bray–Curtis index) was calculated (vegan::vegdist), permutational multivariate analysis of variance (PERMANOVA) was performed (vegan::adonis2), and nonmetric multidimensional scaling (NMDS) ordination was used (vegan:: metaMDS) to plot the data with vegan R package. Linear discriminant analysis effect size (LEfSe) was performed using Mothur [42]. Relative abundance (%) as well as alpha-diversity (Shannon index) was also subjected to the correlation with log_10_(SCC). Spearman correlation and a standard regression model was calculated in R software [43].

### Microbial change analysis

In this study, we focused on the first *S. aureus* CM event from each cow in a new lactation cycle. To compare milk microbial changes in quarters with *S. aureus* CM and healthy quarters, we selected only one healthy quarter from each cow with low SCC (< 200,000 cells/mL) over the whole lactation as a control, except for the one quarter (Q3) of H4C88 due to the overall high SCC in all four quarters in the same cow (Additional file 2: Table S1). One cow (H2C7) was excluded in this specific analysis due to no sequencing result on the first week (Week 0) of *S. aureus* CM. We then compared the milk from mammary quarters that experienced *S. aureus* CM (n = 10) to that of healthy (control) quarters (n = 9). Microbial changes at five time points up to 6 weeks before and 2 weeks after *S. aureus* CM (Week-6, Week-4, Week-2, Week 0, and Week 2) were then analyzed by comparing the milk microbial composition of heathy quarters and *S. aureus* CM quarters. Individual mammary quarters were considered the experimental units used for alpha-diversity, beta-diversity, and LEfSe analysis. We divided *S. aureus* CM cases into two groups (Group I and II) based on the relative abundance (16S rRNA gene amplicon sequencing) of the *Staphylococcus* genus in sick quarters in the first week of the *S. aureus* CM (Additional file 1: Fig. S2). In Group I (14 quarters from 7 cows), the same number of healthy and mastitic milk samples were used in each week: 6 milk samples at both Week-6 and Week-4, 12 samples at Week-2, 14 samples at Week 0, and 8 samples at Week 2. In Group II (6 quarters from 3 cows), we included 6 samples in each week except for Week 2 (4 samples).

### Microbial network analysis

The average rarefied OTU table of all milk samples (n = 583) was used to perform microbial network analysis in R software [43]. Only the 293 OTUs detected at least 10% of the milk samples were included in the analysis to reduce the complexity of the network. The microbial network was analyzed by calculating co-occurrence via Spearman correlation between the OTUs and corroborated with two OTU linear models (GLM), one GLM that included only environmental independent variables and one that included independent variables and relative abundance of each other OTUs [44]. Quasipoisson distribution on the 16S rRNA abundance data for each OTU-OTU combination was used for GLM analysis. Sample source (cow and quarter) was considered as an independent variable. Correlations between two OTUs were filtered by *p*-value (< 0.01) in both analyses [44, 45]. Potential false positive or negative interactions indicated by non-corroborated results from the Spearman analysis and the GLM analysis were excluded in further analysis. The interactions where the Spearman's ρ was ≥ 0.2 or ≤ − 0.2 were included, which was then visualized using Cytoscape (v. 3.8.2) by the β [46].

### Shotgun metagenomic sequencing data analysis

The metagenomic DNA generated 50.6 million reads in average per sample. The resulting FASTQ files were processed to trim low-quality bases for a cut-off value of 20 and adaptors and host-specific reads were removed using the ReadQC module of metaWRAP (v. 1.2.1) [47]. Bos taurus 3.1 (UMD 3.1, https://bovinegenome.elsiklab.missouri.edu/downloads/UMD_3.1) was used as a reference genome to remove the host-specific reads. The resulting reads were 717,369 (190507C7Q2), 166,400 (190923C74Q1), and 649,838 (649,838). The cleaned reads were assembled and then used to analyze microbial community at species using Kraken2 with miniKraken database [48]. The cleaned reads were also assembled with the metaSPAdes (v. 3.15.2) and then classified into taxonomic bins using CONCOCT (v.1.0.0), MaxBIN2 (v. 2.2.6), and metaBAT2 (v. 2.2.15) [49–52]. The classified bins were processed to reduce contamination through RefineM (v. 0.1.2), and the refined bins were then evaluated with CheckM (v. 1.1.3) [53, 54]. Following the bin refinement, metagenome-assembled genomes (MAGs) were processed in Prokka (v. 1.14.5) to annotate the encoded genes [55]. Virulence genes and antimicrobial resistant genes were analyzed using ABRicate (https://github.com/tseemann/abricate) through the VFDB database. BAGEL4, AntiSMASH, and KEGG were used to find bacteriocin, secondary metabolites, and metabolic pathways [56–58].

## Results

In this study, 19.3% (135/698) of the dairy cattle were affected by CM, of these infections, only 7.4% (10/135) were caused by *S. aureus*; the other infections were caused by various etiological agents such as non-aureus Staphylococci (NAS), *Escherichia coli*, or *Klebsiella pneumoniae*. The first *S. aureus* CM cases from each cow occurred between 8 and 203 days of milk (DIM): three in transition (1–21 DIM), two in early lactation (22–100 DIM), four in mid lactation (101–200 DIM), and one in late lactation (> 201 DIM). A total of 599 milk samples from all four quarters were collected from 10 Holstein dairy cows diagnosed with *S. aureus* CM during the 15-month study period. Among those, sequencing failed on six samples due to low bacterial DNA concentration. A total of 13,854,684 sequence reads passed filter with an average count of 22,418 sequence reads per sample, including milk samples (n = 593) and controls (n = 25). During rarefication, ten milk samples were removed due to low library size, leaving 583 milk samples for further analysis. None of the negative controls included in PCR reactions resulted in visible PCR bands on gel electrophoresis and cross-contamination of the negative and positive controls were not recognized. Thus, OTUs derived from controls were not removed from the sample dataset.

### Overall microbiota across cows during sampling period

**Fig. 1:**
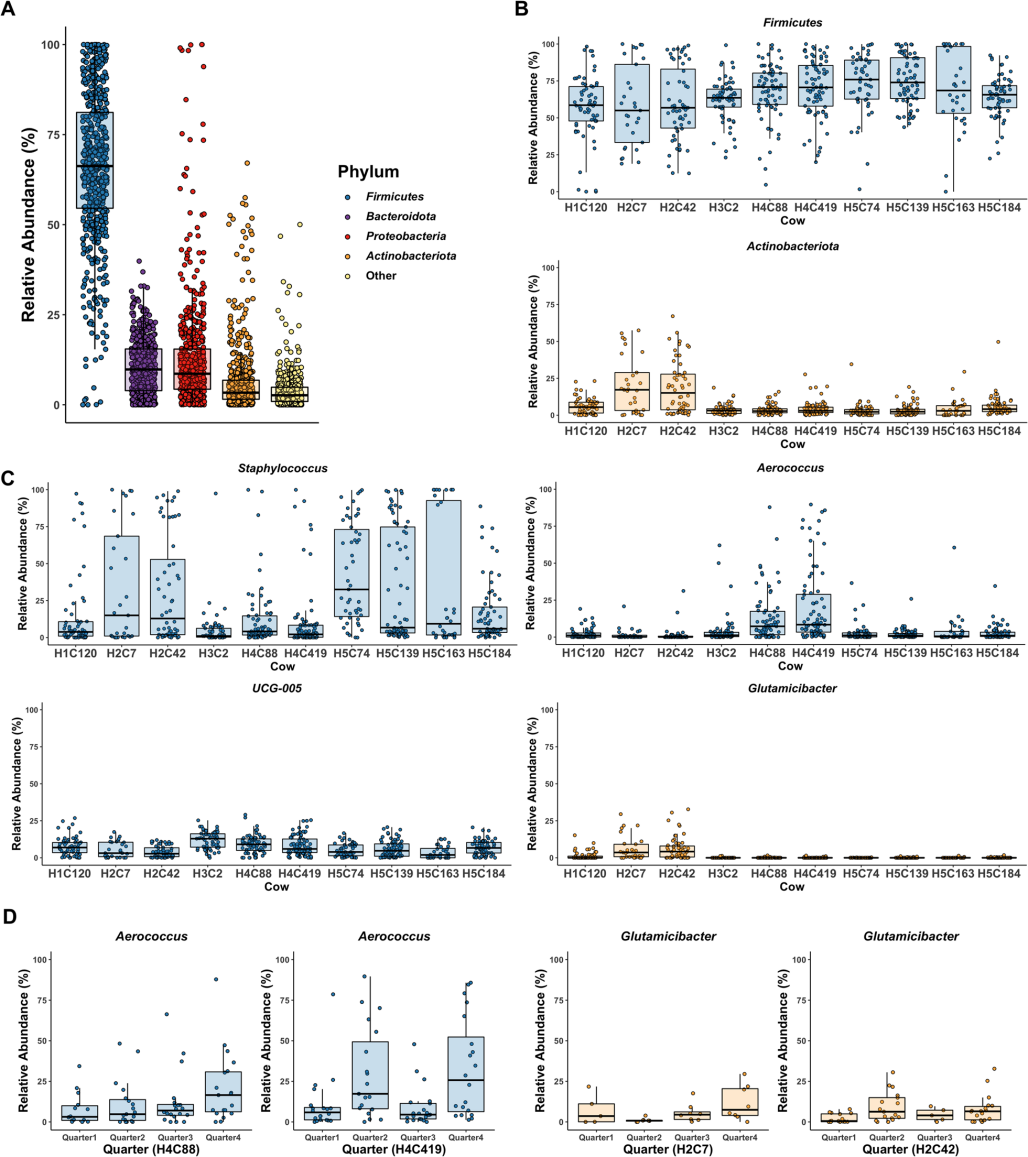
Relative abundance of the raw milk microbiota of ten cows associated with *S. aureus* clinical mastitis. **A** The relative abundance of each phylum in the milk samples showed four majority phyla: *Firmicutes*, *Bacteroidota, Proteobacteria,* and *Actinobacteriota*. **B** The relative abundance of *Firmicutes* and *Actinobacteriota* varied in ten cows. **C** At the genus level, *Staphylococcus* was the most abundant genus in all ten cows. The distribution of *Aerococcus* was high in H4C88 and H4C419 and *Glutamicibacter* was more abundant in H2C7 and H2C42 in other cows. **D** The relative abundance of *Aerococcus* and *Glutamicibacter* differed at the quarter level. Identifiers of each cow name are ‘H’ for the herd and ‘C’ for cow number

Taxonomic profile analysis with all milk samples showed bacterial phyla with different relative abundance were shared by the five herds. *Firmicutes* was predominant with an average relative abundance of 65.7% followed by *Bacteroidota*, *Proteobacteria*, and *Actinobacteriota* (Fig. 1A). *Firmicutes* was highly prevalent across cows during the sampling period. Notably, the average relative abundance of *Aerococcus* was higher than *Staphylococcus* in H4C88 and H4C419 while *Actinobacteriota*, mainly *Glutamicibacter*, was more abundant in H2C7 and H2C42 (Fig. 1B). Within H4C88 and H4C419, differences in the relative abundance of *Aerococcus* were observed at the quarter level with higher abundance in one or two quarters (Fig. 1D). Similarly, *Glutamicibacter* was more abundant in one quarter compared to adjacent quarters in H2C7 and H2C42. The variations in the relative abundances of major phyla and genera in each cow contributed to a cow/quarter-specific microbial community.

### Microbial changes before, during, and after *S. aureus* CM

*S. aureus* CM cases, where *S. aureus* was isolated from the milk collected from the quarters in the first week of CM (Week 0), showed differences in the relative abundance of the *Staphylococcus* genus. This led us to divide *S. aureus* CM cases into two groups. Group I was composed of seven cows (63.6%, 7/11) with the relatively high *Staphylococcus* at Week 0 of infection. Group II was consisted of three cows where the relative abundance of *Staphylococcus* was extremely low (< 10%) at the diagnosis of infection.

**Fig. 2:**
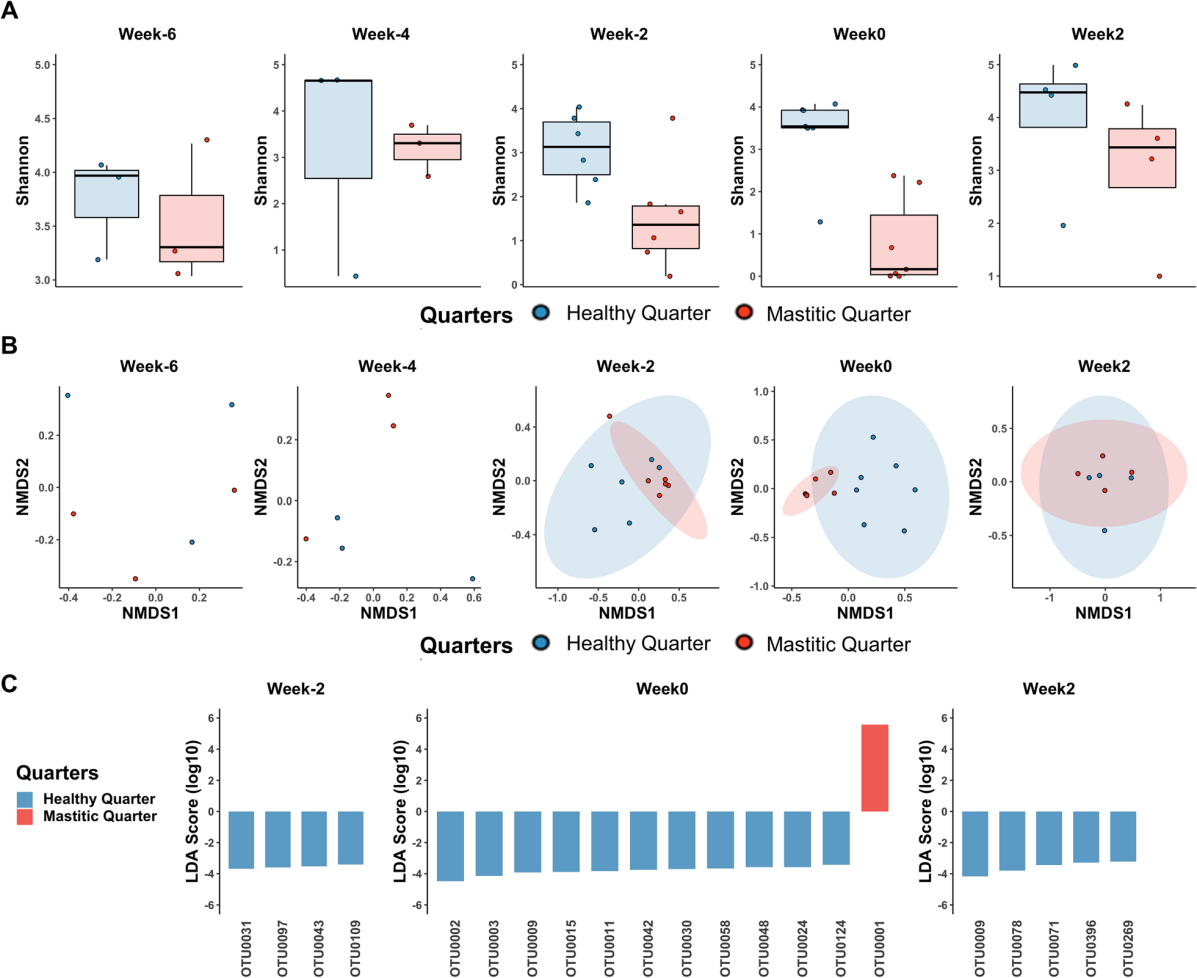
Microbial changes in alpha- and beta-diversity and biomarkers before, during, and after *S. aureus* clinical mastitis (CM) in Group I. Group I consisted of sick cows (n = 7) where *Staphylococcus* was detected in CM quarters at the time of CM diagnosis (Week 0). Both alpha-diversity (**A**) and beta-diversity (**B**) were similar between healthy and mastitic quarters at Week-6 and Week-4. Alpha-diversity in healthy quarters was significantly different from sick quarters two weeks before diagnosis (Week-2). Both alpha- and beta-diversity were significantly dissimilar between healthy and sick quarters at Week 0, which then remained as such while the mastitis continued for a few more weeks. Two weeks after the resolution of *S. aureus* CM (Week2), alpha- and beta-diversity had recovered, and were similar to the healthy quarters. **C** LEfSe analysis showed that at the time of diagnosis OTU0002 (*Aerococcus*) and OTU0001 (*Staphylococcus*) were highly associated with healthy and mastitic quarters, respectively. *Ruminococcaceae* unclassified (OTU0009) was highly associated with healthy quarters at Week 0 and Week 2

In Group I animals (n = 7), up to four weeks prior to *S. aureus* CM (Week-4), both alpha-diversity and beta-diversity in the healthy quarters and the future CM quarters were not significantly different (Fig. 2). LEfSe analysis was also unable to identify any specific OTU correlated with either the healthy or the future CM quarters at Week-6 and Week-4. Differences in the microbial profiles were observed starting two weeks before *S. aureus* CM (Week-2). The alpha-diversity in the future CM quarters at Week-2 was significantly different between healthy and future CM quarters (Shannon *p* < 0.05; Mann–Whitney statistic 32) although Chao1 and Simpson indices showed no significant difference (Additional file 3: Table S2). PERMANOVA analysis of the Bray–Curtis dissimilarities revealed that the beta-diversity at Week-2 was highly dissimilar between healthy and future CM quarters (PERMANOVA *p* < 0.05, *F* = 2.32). LEfSe identified 4 OTUs that were highly associated with healthy quarters at Week-2. In the first week of *S. aureus* CM (Week 0), both alpha- and beta-diversity in the sick quarters were significantly distinguished from the healthy quarters (Shannon *p* < 0.05; Mann–Whitney statistic 47; PERMANOVA *p* < 0.05, *F* = 7.07). LEfSe identified 12 differentially abundant OTUs, yet none of them was overlapped with those found at Week-2. OTU0002 (LDA score = − 4.47, *p* = 0.03) and OTU0001 (LDA score = 5.58, *p* = 0.002) were highly associated with healthy and mastitic quarters, respectively. Two weeks after *S. aureus* CM (Week 2), both alpha- and beta-diversity in the infected quarters were indistinguishable from the healthy quarters suggesting the re-establishment of the microbiota. However, five OTUs were still significantly more abundant in the healthy quarters and OTU0009 corresponding to *Ruminococcaceae* unclassified was found in healthy quarters at Week 0 (LDA score = − 3.93, *p* = 0.008) and Week 2 (LDA score = − 4.17, *p* = 0.04) consecutively.

Group II animals consisted of cows (n = 3) where the relative abundance of *Staphylococcus* was barely detected at Week 0 in CM milk samples (191119C120Q2, 190805C419Q4, and 191118C184Q4). Those milk samples were initially diagnosed with *S. aureus* CM because *S. aureus* was isolated from mastitic milk samples using microbiological culture. From those CM milk samples in Group II, we found that other bacteria were sometimes isolated concurrently with *S. aureus* (Additional file 4: Table S3). Two more phenotypically different colonies with no hemolytic activity on blood agar were isolated with *S. aureus* (1 CFU/0.01 mL of milk) from 191119C120Q2. *Corynebacterium bovis* (4 CFU/0.01 mL of milk) and *S. aureus* (1 CFU/0.01 mL of milk) were isolated from 190805C419Q4, yet the abundance of *Corynebacterium* was not detected in 190805C419Q4. *Aerococcus viridans* (10 CFU/0.01 mL of milk) and *S. aureus* (10 CFU/0.01 mL of milk) were isolated from 191118C184Q4, and we confirmed that the relative abundance of *Aerococcus* was higher than 34% in 191118C184Q4. To note, the sick quarters of H4C419 and H5C184 experienced CM caused by other etiological agents at Week-2. Interestingly, the alpha- and beta-diversity of three cows in Group II was not significantly dissimilar between healthy and sick quarters at all time-points from Week-6 to Week 2 (Additional file 3: Table S2). LEfSe identified that 14 OTUs highly associated with either healthy or mastitic quarters before and during *S. aureus* CM. Among them, OTU0001 (*Staphylococcus*) was significantly associated with healthy quarters (LDA score = − 5.28, *p* = 0.05) (Additional file 3: Table S2).

### Network analysis of the microbial community

**Fig. 3:**
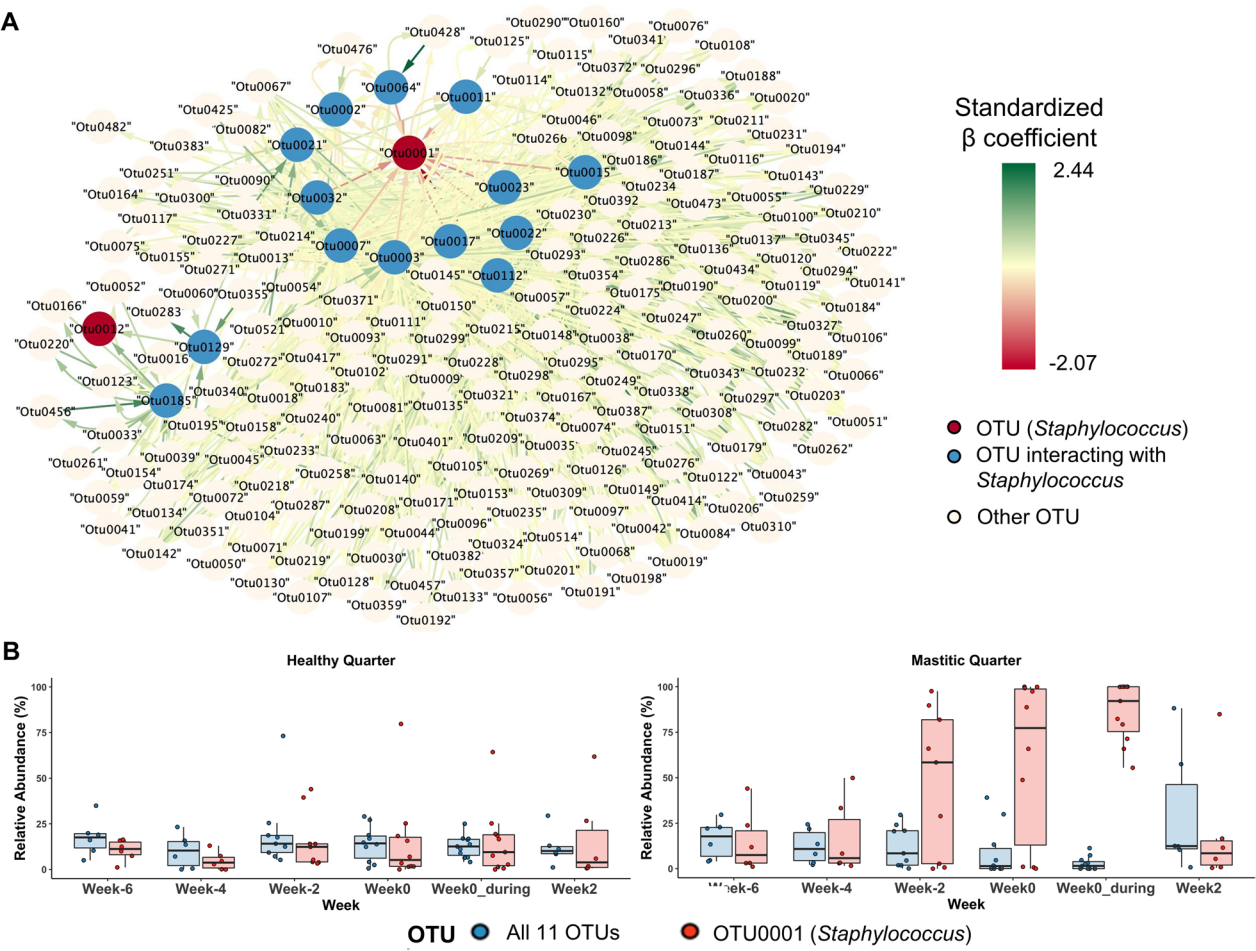
Microbial network analysis of the milk microbiota and their relative abundance. **A** The network is based on the combination of classical Spearman correlation-based network analysis corroborated with a GLM approach. Each node represents a taxonomic group at the OTU level. Arrows depict the direction of the relationship (source to target) based on the β calculation from GLM analysis. Green and red connections indicate the relative strength of the positive and negative relationships, respectively. Only 1242 interactions where 16 OTUs including two OTUs corresponding to *Staphylococcus* were involved were shown. **B** The relative abundance of two groups (11 OTUs and OTU0001) over 8-week period including before, during, and after *S. aureus* CM was compared in healthy quarters (n = 10) and quarters with clinical mastitis (n = 11)

Out of 85,837 possible species interactions (293^2^ − 12, self-interactions excluded), only 5561 interactions involving 278 OTUs were left after filtering (Additional file 5: Table S4). Among them, two OTUs (OTU0001 and OTU0012) corresponding to *Staphylococcus* were involved in 25 interactions with 14 OTUs (Fig. 3A). All interactions between OTU0001 and other OTUs were bi-directional except for OTU0021 and the interactions between OTU0012 and two other OTUs were one-direction. The β in GLM analysis showed that all 11 OTUs had stronger negative impacts on OTU0001 (*Staphylococcus*) than the reciprocal effects. However, the relationships between OTU0001 and 11 OTUs were negligible (Spearman's ρ > − 0.2). Only two OTUs corresponding to UCG-005 and *Aerococcus* had moderate (Spearman's ρ > − 0.4) and weak (Spearman's ρ > − 0.3) interactions with OTU0001, respectively. For a stricter analysis we excluded all samples (n = 45) where colonies with more than three phenotypes were isolated on blood agar. This analysis also identified the same OTUs as having a negative impact on OTU0001, except for OTU0022 (*Ruminobacter*) which was identified in the first analysis but was not identified in the second more stringent analysis. We further compared the relative abundance of 11 OTUs collectively with OTU0001 (Fig. 3B). The relative abundance of 11 OTUs in healthy quarters was consistently higher than OTU0001 before, during, and after *S. aureus* CM dramatically varied in CM quarters. The relative abundance of 11 genera as a group was higher than OTU0002 in *S. aureus* CM quarters at Week-6 and Week-4, became lower than OTU0001 at Week-2 and Week 0. This difference in the relative abundance between 11 OTUs and OTU0001 became more obvious during consecutive weeks while *S. aureus* CM continued for few more weeks (Week0_during), and then decreased as before *S. aureus* CM at Week2.

### Relationship between SCC and milk microbiota

**Fig. 4:**
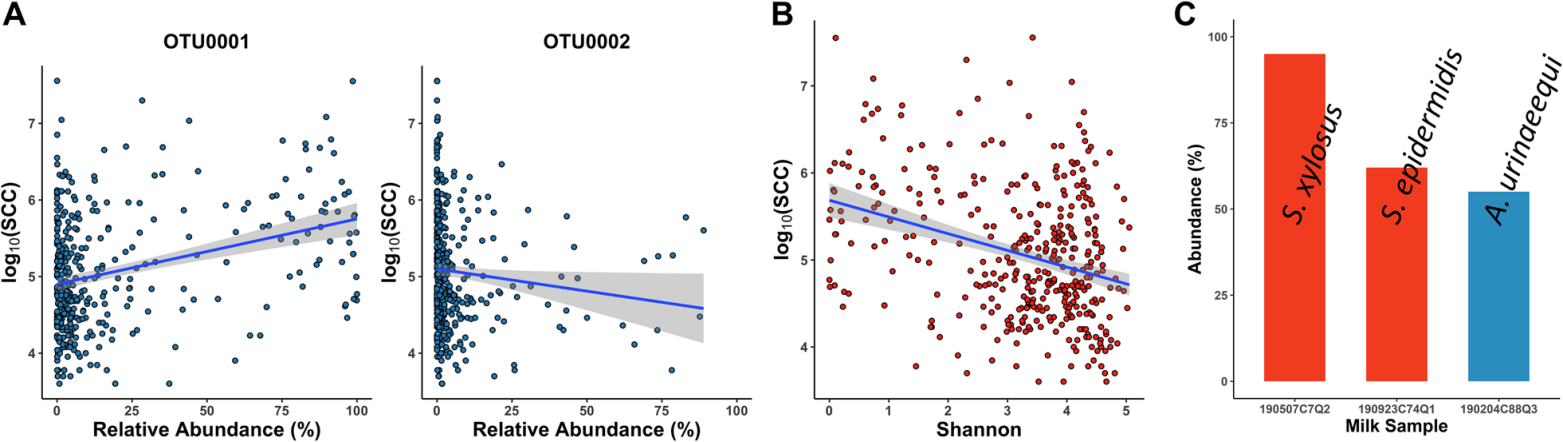
Relative abundance of milk microbiota. **A** OTU0001 and OTU0002 were predominant in milk samples with low SCC. **B** Shannon index was negatively correlated with log_10_(SCC) (Spearman's ρ > − 0.3). **C** Shotgun metagenomic sequencing identified the bacterial species in healthy milk samples and revealed the relative abundance (%)

The range of SCC in this study was from 4000 cells/mL to 35,891,000 cells/mL in non-CM milk (n = 385). The correlation between the relative abundance of each OTU (n = 5068) and log_10_(SCC) was either negligible or weak. Interestingly, OTU0001 (*Staphylococcus*) and OTU0002 (*Aerococcus*) were observed at relatively high abundance in several milk samples with low SCC (< 200,000 cells/mL) (Fig. 4A). For instance, high abundance of single OTU was found in milk samples: 190507C7Q2 (OTU0001, 100%), 190923C74Q1 (OTU0001, 86.5%) and 190204C88Q3 (OTU0002, 37%). The correlation between these two OTUs and log_10_(SCC) were weak and the directions were opposite. To examine the relationship between bacterial diversity and inflammation, we further analyzed the correlation between Shannon index and log_10_(SCC). Shannon index was negatively correlated with log_10_(SCC), but it was weak (Spearman's ρ > − 0.3) (Fig. 4B). This week correlation was also observed in the analysis that excluded milk samples that produced > 3 types of colony morphology on blood agar (n = 45).

### *Staphylococcus *spp. and *Aerococcus* spp. in healthy milk

To further explore what species of OTU0001 (*Staphylococcus*) and OTU0002 (*Aerococcus*) were present in healthy milk samples, we performed shotgun metagenomic sequencing on three milk samples: 190507C7Q2 (SCC = 59,000 cells/mL), 190923C74Q1 (SCC = 143,000 cells/mL), and 190204C88Q3 (SCC = 43,000 cells/mL). Among them, sample 190204C88Q3 produced > 3 different phenotypes on blood agar. The bacterial contigs were 543,451 (190507C7Q2), 9747 (190923C74Q1), and 107,032 (190204C88Q3) due to a tremendous amount of host DNA contamination. Kraken2 analysis showed the majority portion of bacterial species in these milk samples were *S. xylosus* (95%, 190507C7Q2), *S. epidermidis* (62%, 190923C74Q1), and *A. urinaeequi* (55%, 190204C88Q3) (Fig. 4C and Additional file 6: Table S5). Of these, we were able to reconstruct two MAGs from the taxonomic bins with good quality (> 85% completeness, < 2% contamination). These MAGs were identified as *S. xylosus* and *A. urinaeequi* with the genome size of 2.3 Mb (CDS 2234) and 1.5 Mb (CDS 1373), respectively. No known virulence genes were found from either *S. xylosus* MAG or *A. urinaeequi* MAG. An antimicrobial resistance gene was only found from *S. xylosus* MAG, which was associated with aminoglycosides resistance (K19299). AntiSMASH of *S. xylosus* MAG showed five gene clusters associated with secondary metabolites including staphyloferrin A (K23447, K23446, and K21898), staphyloxanthin (K10208, K10209, K10210, and K10211), and squalene (K00801). However, *A. urinaeequi* MAG showed no predicted gene cluster in the final bin; although, a gene cluster for lycopene biosynthesis (K02291 and K10027) was found in pre-refined bins.

## Discussion

In this study, we investigated the milk microbiota before, during, and after *S. aureus* CM by tracking the health status of all four quarters in 10 dairy cows that developed *S. aureus* mastitis during the 15-month study period. We performed 16S rRNA gene amplicon sequencing on all samples and shotgun metagenomics on three samples from healthy control quarters. This longitudinal cohort study on the milk microbiota allows us to study microbial changes associated with cows experiencing a natural *S. aureus* CM – rather than cows infected in an artificial challenge model.

The composition of the host microbiome is different across body sites, time, and health status. In dairy cattle, prior microbiota studies have focused on the microbial profiles of different niches, the differences between CM quarters and healthy quarters at the same time point, and microbial alteration in response to mastitis treatments [27, 28, 59]. However, farm-to-farm variation in the microbial composition could lead to discrepancies between milk microbiome studies. Several studies have shown that each farm is a particular niche with its own persistent microbiota [60, 61]. In this study, we observed that composition of milk microbiota varied at the cow level as well as the quarter level. These variations were likely due to the environment or infections, yet we failed to prove the herd-specificity due to a limited and uneven number of cows from each herd included in this study. Cow/quarter-specific microbiota and its variations challenge the milk quality control and the development of the early microbial detection method for bovine mastitis using microbial indicators. Indeed, the relative abundance of all biomarkers (OTUs) in this study identified by LEfSe analysis was inconsistent during the study period, suggesting no single taxon able to represent microbial health in bovine intramammary glands. However, the relative abundance of 11 OTUs as a group was detectable over the lactation (Additional file 1: Fig. S3).

In this study, we only considered the first *S. aureus* CM in each quarter during a new lactation cycle to investigate the microbial changes before, during, and after *S. aureus* CM. Andrews et al. previously reported that the milk microbiome of infected quarters was frequently dominated by a single OTU among milk sampled collected from 28 infected quarters [27]. In the first week of the *S. aureus* CM, OTU0001 was predominant with the relative abundance of higher than 80% in five milk samples. We also observed the relative abundance of OTU0001 was high (> 80%) in three milk samples two weeks prior to the *S. aureus* CM being noted by the producer or research staff via visual inspection. SCC of the milk samples two weeks prior to *S. aureus* CM being diagnosed was between 57,000 cells/mL and 12,107,000 cells/mL, indicating largely different stages of the intramammary infections. This difference may result from the pathogenicity of *S. aureus*, the resistance/tolerance of the resident microbiota to *S. aureus*, or the immune response mounted by a particular animal [62–64]. It may be simply because of different time gaps between *S. aureus* colonization and subsequent inflammation of the mammary glands [65]. However, due to the unavailability of data on the exact starting date of *S. aureus* CM, the time gaps between two weeks prior and the actual onset of CM could not be determined. We also observed that the microbiota in sick quarters recovered and resembled the microbiota of healthy quarters within 2 weeks after *S. aureus* CM. This result agrees with the previous studies conducted by Ganda et al. showing the re-establishment of milk microbiome of the CM quarters within 14 days via natural infection and experimental infection of Gram-negative pathogens [28, 30].

Unexpectedly, we also found *S. aureus* CM cases where *Staphylococcus* was barely detected at Week 0 although *S. aureus* was isolated from the same milk samples. This discordance has been rarely reported previously probably due to the insufficient sequencing depth unable to detect rare members of the microbiota [66–68]. This may result from other intrinsic factors and extrinsic factors we could not detect or notice. It is worth emphasizing that there was another CM infection in the same quarter right before *S. aureus* CM in Group II, which might overshadow *Staphylococcus*. At Week0 in Group II, more interestingly, *Staphylococcus* (OTU0001) was significantly associated with the healthy quarters (Additional file 3: Table S2). Considering the low SCC (15,000 cells/mL to 282,000 cells/mL), *Staphylococcus* in these healthy quarters was likely to be NAS. This finding suggests that high alpha-diversity neither represent microbial resilience against nor susceptibility to  pathogenic bacteria, nor is associated with healthy outcome in bovine intramammary glands.

From network analysis, we observed that 11 OTUs had negative interactions with the relative abundance of OTU0001 (*Staphylococcus*). This correlation is likely observed since *S. aureus* displaces many of these OTUs as it becomes a dominant member of the microbiome during infection. Each of 11 these OTUs comprised a minor population at different time points, yet each were commonly found in milk samples we analyzed (32% to 91%). Beside the interactions between these 11 OTUs with OTU0001, they had positive impacts on each other and many other OTUs (n = 202). This intertwined microbiota could provide the microbial resilience to pathogen colonization collectively and allow variations of an individual group in the udder. This result suggests that bacteria in bovine mammary glands may collaborate and serve as healthy microbiota and offer cross-protection against mastitis pathogens in different manners directly and indirectly.

We also investigated the relationship between SCC and bacterial abundance. Although there was no specific taxon strongly correlated with log_10_(SCC) in this study, we found two OTUs either *Staphylococcus* or *Aerococcus* was highly predominant in some milk samples from the healthy quarters with low SCC (< 200,000 cells/mL), which indicates no pro-inflammatory activity caused by these OTUs present in those specific milk samples. This finding is interesting because the significantly decreased microbial diversity generally implies an imbalanced microbiota, which tends to be more vulnerable to incoming or pathogenic bacteria [69, 70]. However, in this study, we observed a single taxon was predominant without triggering host immune response. If this specific group of bacteria are also equipped with antagonizing ability toward mastitis pathogens, they could be promising candidates for bovine intramammary probiotics. Shotgun metagenomic sequencing revealed that *S. xylosus*, *S. epidermidis*, and *A. urinaeequi* were highly predominant in three different healthy milk samples and represented 95%, 62%, and 55% of the bacterial population in each sample. Subclinical or milk clinical mastitis can be caused by NAS, such as *S. xylosus* and *S. epidermidis*, and they are often isolated from quarters with low SCC as well as high SCC [71, 72]. *S. xylosus* is known to interfere with the *S. aureus agr* quorum-sensing system and inhibit the biofilm formation ability of *S. aureus* [73, 74]. *S. epidermidis* is a well-known antagonistic bacterium against *S. aureus* in biofilm formation, growth, and quorum-sensing [75–77]. Thus, NAS which are not associated with strong intramammary inflammation, such as those observed in samples 190507C7Q2 and 190923C74Q1, have the potential to be developed into anti-*S. aureus* probiotics. Unlike NAS, *A. urinaeequi* has been scarcely studied and its antimicrobial activity has been previously identified against only Gram-negative bacteria [78]. However, our group has previously reported that *A. urinaeequi* strain isolated from dairy milk was able to inhibit intramammary gland infection-associated *S. aureus* strains in co-culture conditions [79], making this genus also of interest for the development of anti-*S. aureus* probiotics.

In this microbiome study, we characterized the microbial community and its changes before, during, and after *S. aureus* CM in dairy cows and identified bacterial interaction that may play an important role in udder health. We also identified bacterial interactions where 11 OTUs or possibly more were negatively involved with *Staphylococcus* (OTU0001) and may be associated with the susceptibility to *S. aureus* CM. We also provide evidence that unbalanced milk microbiota caused by a certain group of bacteria was not always associated with disease or inflammation. Our findings are suggestive of a potential application of microbial modulation and perturbation in bovine udder to prevent future instances of bovine mastitis using a group of bacteria that antagonizes pathogens but induces no strong inflammation. However, a limited number of *S. aureus* CM cases and herds may result in biological and geographical bias in this study. Furthermore, identification of the abundant bacterial species from a limited number of milk samples may lead to misinterpretation of the potential anti-*S. aureus* probiotics. Therefore, further studies need to focus on the antagonistic interactions between *S. aureus* and potential probiotics as well as their pro-inflammatory effects in vivo and in vitro.

### Supplementary Information

**Additional file 1: Fig. S1.** Holstein cows with *S. aureus* clinical mastitis. This diagram illustrates 10 dairy cows diagnosed with *S. aureus* clinical mastitis from five dairy herds in Quebec, Canada. All mastitis occurred via natural infections. Milk samples (n = 599) were collected from all four quarters bi-weekly. Identifiers of each cow name are ‘H’ for the herd and ‘C’ for the assigned cow number. The red and the blue square boxes represent clinical mastitis with visible symptoms infected by *S. aureus* and other mastitis pathogens, respectively. The black square boxes represent non-mastitic milk. The open square boxes regardless of colors indicate milk samples where no 16S rRNA data is available due to missing milk samples (n = 16), low bacterial DNA (n = 6), and low library read size (n = 10). **Fig. S2** Groups of dairy cows. A total of ten dairy cows (11 quarters) affected by *S. aureus* clinical mastitis were grouped based on the relative abundance of *Staphylococcus* at Week 0. Two quarters, a healthy and a CM quarter, were selected and indicated under the name of each cow. At Week 0, *Staphylococcus* was solely the predominant genus in mastitic milk samples in Group I while it was barely detectable in Group II with relative abundance of less than 10%. **Fig. S3** Relative abundance of 11 OTUs and OTU0001 (Staphylococcus) over the lactation in healthy and mastitic quarters. The line graphs depict the changes of the relative abundance of 11 OTUs and OTU0001 in two quarters (healthy vs. mastitic quarters) from each cow over the study period. The vertical dotted lines indicate either beginning or ending of *S. aureus* CM.

**Additional file 2: Table S1.** Metadata of all milk samples (n = 583) collected from the cows diagnosed with *S. aureus* clinical mastitis

**Additional file 3: Table S2.** Microbial changes before, during, and after *S. aureus* clinical mastitis in Group I and Group II

**Additional file 4: Table S3.** Etiological agents isolated from clinical mastitic milk samples

**Additional file 5: Table S4.** Microbial network analysis in milk samples (n = 583)

**Additional file 6: Table S5.** Bacterial species/strains and their relative abundance (%) found in three milk samples from the shotgun metagenomic sequneces

## Data Availability

All sequencing data is available through the NCBI Sequence Read Archive (SRA) under BioProject identifiers PRJNA752361.
