# Correction to: A longitudinal census of the bacterial community in raw milk correlated with *Staphylococcus aureus* clinical mastitis infections in dairy cattle

**DOI:** 10.1186/s42523-023-00273-5

**Published:** 2023-10-12

**Authors:** Soyoun Park, Dongyun Jung, Ianina Altshuler, Daryna Kurban, Simon Dufour, Jennifer Ronholm

**Affiliations:** 1 Faculty of Agricultural and Environmental Sciences, Macdonald Campus, McGill University, Montreal, QC Canada; 2 Faculté de Médecine Vétérinaire, Université de Montréal, Saint-Hyacinthe, QC Canada; 3 Mastitis Network, Saint-Hyacinthe, QC Canada; 4 Regroupement FRQNT Op+Lait, Saint-Hyacinthe, QC Canada

**Correction to: Animal Microbiome (2022) 4:59**

10.1186/s42523-022-00211-x

Following publication of the original article [1], we have been notified the one of the authors’ last name was published incorrectly.

It was published as follows:

Daryna Kruban

It should be as per below:

Daryna Kurban

The original article was updated.
