# Supplementary material for: A longitudinal census of the bacterial community in raw milk correlated with Staphylococcus aureus clinical mastitis infections in dairy cattle
Source: Anim Microbiome. 2022 Nov 24;4:59. doi: 10.1186/s42523-022-00211-x (PMC9701008; doi:10.1186/s42523-022-00211-x)
Supplement: Supplementary file 1 — Additional file 1: Fig. S1. Holstein cows with S. aureus clinical mastitis. This diagram illustrates 10 dairy cows diagnosed with S. aureus clinical mastitis from five dairy herds in Quebec, Canada. All mastitis occurred via natural infections. Milk samples (n = 599) were collected from all four quarters bi-weekly. Identifiers of each cow name are ‘H’ for the herd and ‘C’ for the assigned cow number. The red and the blue square boxes represent clinical mastitis with visible symptoms infected by S. aureus and other mastitis pathogens, respectively. The black square boxes represent non-mastitic milk. The open square boxes regardless of colors indicate milk samples where no 16S rRNA data is available due to missing milk samples (n = 16), low bacterial DNA (n = 6), and low library read size (n = 10). Fig. S2 Groups of dairy cows. A total of ten dairy cows (11 quarters) affected by S. aureus clinical mastitis were grouped based on the relative abundance of Staphylococcus at Week 0. Two quarters, a healthy and a CM quarter, were selected and indicated under the name of each cow. At Week 0, Staphylococcus was solely the predominant genus in mastitic milk samples in Group I while it was barely detectable in Group II with relative abundance of less than 10%. Fig. S3 Relative abundance of 11 OTUs and OTU0001 (Staphylococcus) over the lactation in healthy and mastitic quarters. The line graphs depict the changes of the relative abundance of 11 OTUs and OTU0001 in two quarters (healthy vs. mastitic quarters) from each cow over the study period. The vertical dotted lines indicate either beginning or ending of S. aureus CM. [file 42523_2022_211_MOESM1_ESM.docx]

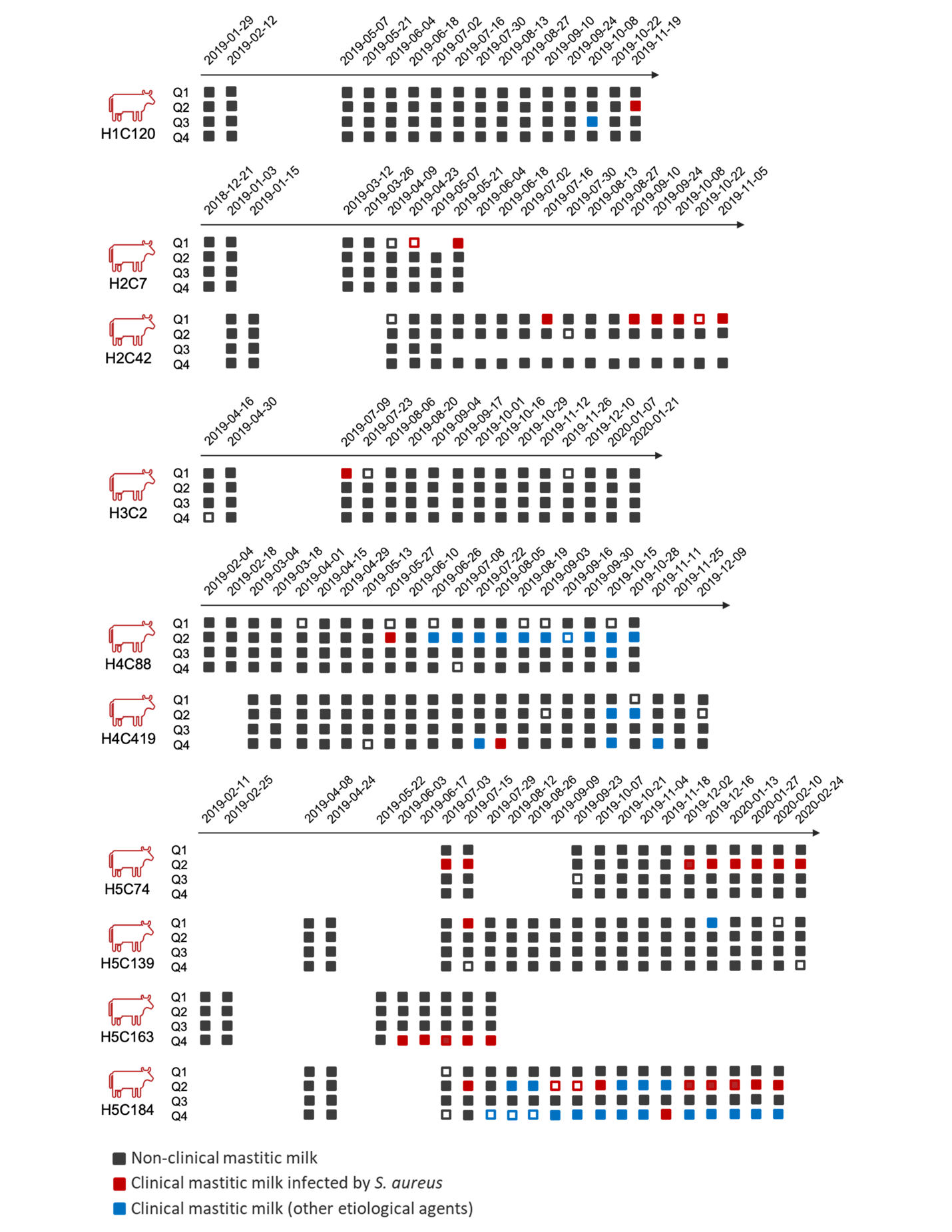


**Fig. S1 Holstein cows with S. aureus clinical mastitis.** This diagram illustrates 10 dairy cows diagnosed with S. aureus clinical mastitis from five dairy herds in Quebec, Canada. All mastitis occurred via natural infections. Milk samples (n = 599) were collected from all four quarters bi-weekly. Identifiers of each cow name are ‘H’ for the herd and ‘C’ for the assigned cow number. The red and the blue square boxes represent clinical mastitis with visible symptoms infected by S. aureus and other mastitis pathogens, respectively. The black square boxes represent non-mastitic milk. The open square boxes regardless of colors indicate milk samples with no 16S rRNA data available due to missing samples (n = 16), low bacterial DNA (n = 6), and low library read size (n = 10).


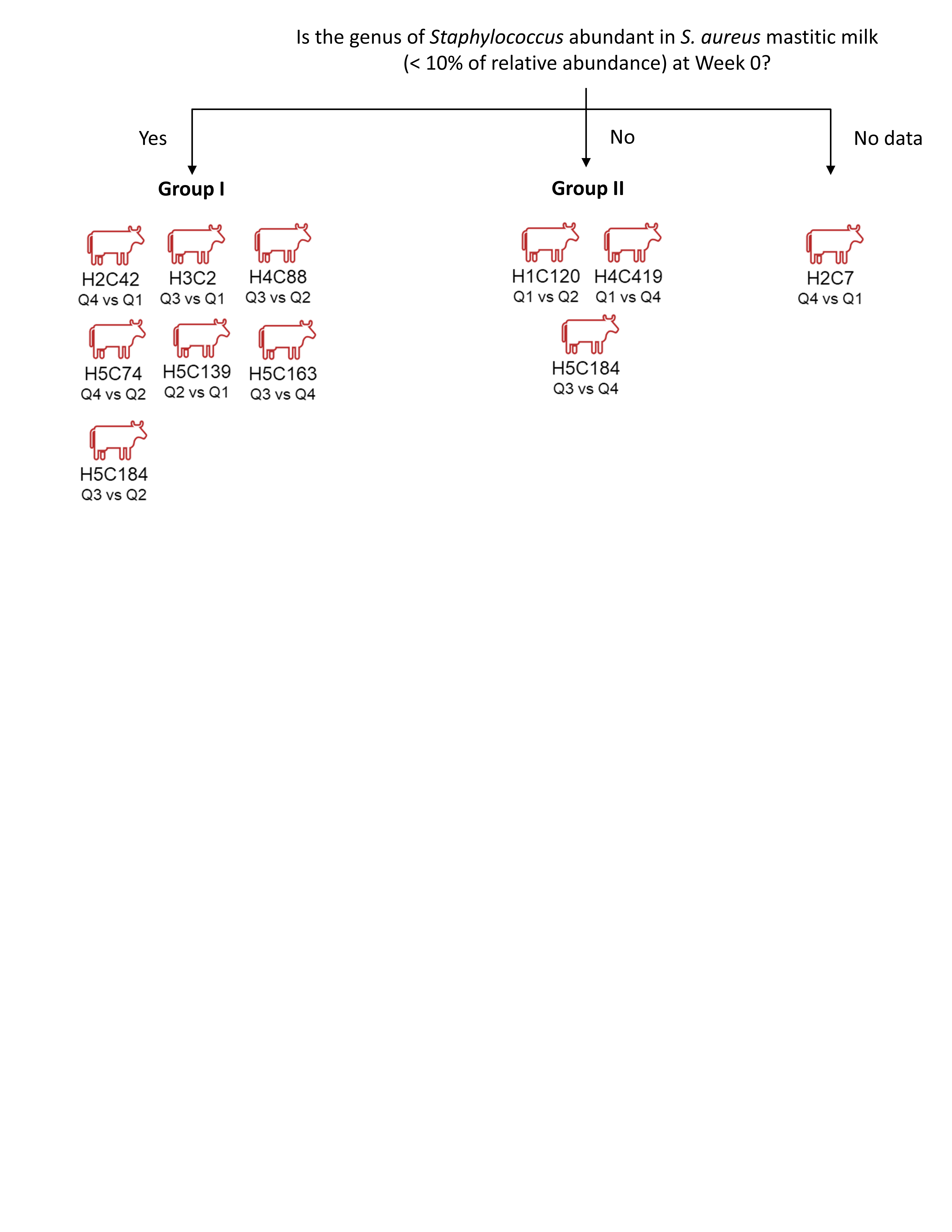


**Fig. S2 Groups of dairy cows.** A total of ten dairy cows (11 quarters) affected by S. aureus clinical mastitis were grouped based on the relative abundance of Staphylococcus at Week 0. Two quarters, a healthy quarter and a CM quarter, were selected and indicated under the name of each cow. At Week 0, Staphylococcus was solely the predominant genus in mastitic milk samples in Group I while it was not detectable in Group II.


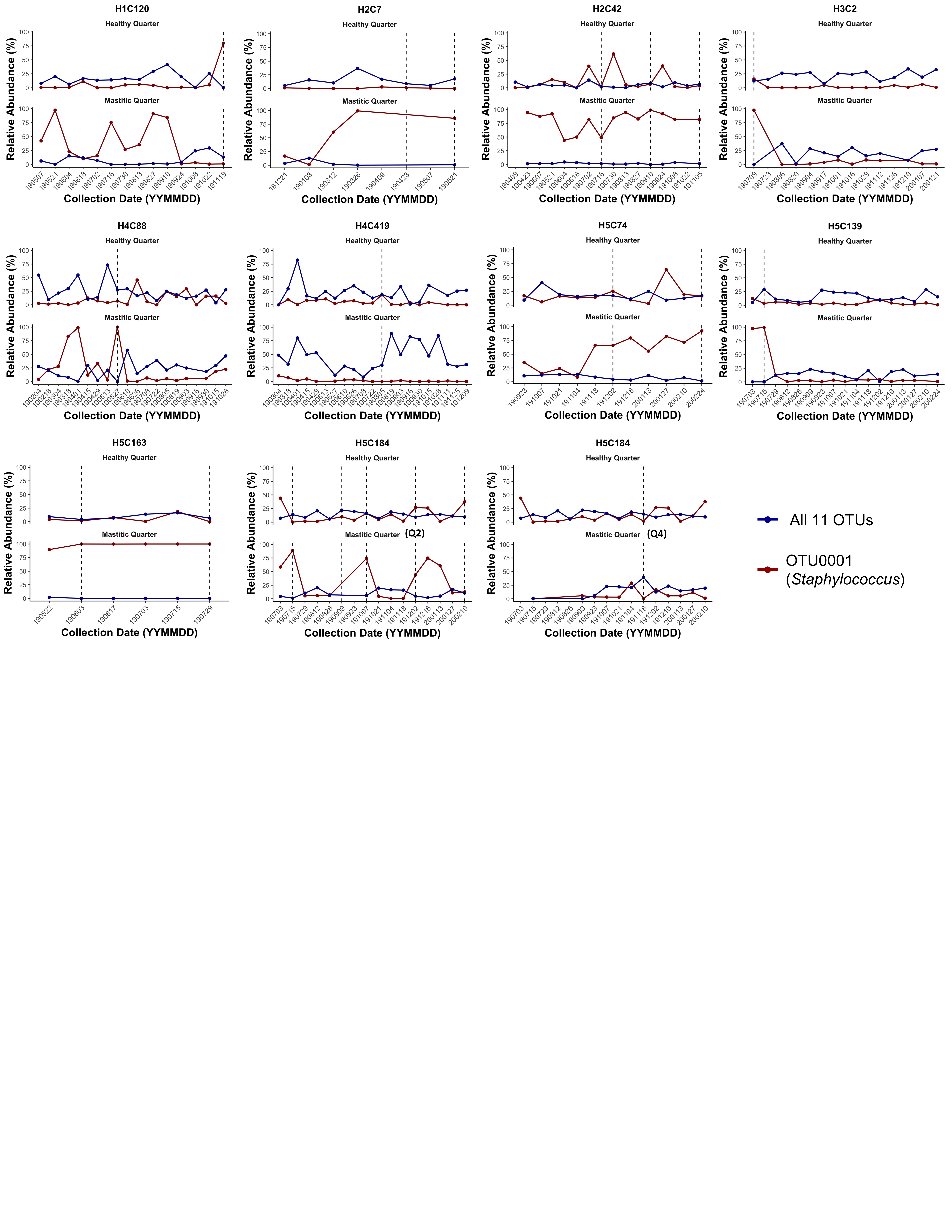


**Fig. S3 Relative abundance of 11 OTUs and OTU0001 (*Staphylococcus*) over the lactation in healthy and mastitic quarters.** The line graphs depict the changes of the relative abundance of 11 OTUs and OTU0001 in two quarters (healthy vs. mastitic quarters) from each cow over the study period. The vertical dotted lines indicate either beginning or ending of *S. aureus* CM.
